# Reviewing ecosystems of evidence: synthesising the evidence on the commercial determinants of health from a complex systems perspective

**DOI:** 10.1186/s13643-026-03187-w

**Published:** 2026-05-22

**Authors:** Alice Tompson, James Thomas, Harry Rutter, Laurence Blanchard, Éadaoin Cott, Rebecca E. Glover, Cecile Knai, Nason Maani, May C. I. van Schalkwyk, Vivian Welch, Mark Petticrew

**Affiliations:** 1 Department of Public Health, Environments and Society, Faculty of Public Health and Policy, LSHTM, London, UK; 2 EPPI-Centre, Social Science Research Unit, UCL Institute of Education, University College London, London, UK; 3 Centre for 21st Century Public Health, University of Bath, Bath, UK; 4 Department of Health Services Research and Policy, Faculty of Public Health and Policy, LSHTM, London, UK; 5 Department of Health, University of Bath, Bath, UK; 6 Global Health Policy Unit, School of Social and Political Science, The University of Edinburgh, Edinburgh, UK; 7 Centre for Pesticide Suicide Prevention, University of Edinburgh, Edinburgh, UK; 8 Bruyère Research Institute, University of Ottawa, Ottawa, Canada

**Keywords:** Commercial determinants of health, Methods, Systematic reviews, Complex systems, Systems thinking

## Abstract

The Commercial Determinants of Health (CDoH) are the systems, practices, and pathways through which commercial actors drive health and equity. This includes their influence on systems of evidence production and dissemination (evidence ecosystems) in order to protect and promote commercial interests. In the Commercial Determinants of Health and Evidence Synthesis (CODES) methodological guidance, we provided advice for conducing CDoH relevant evidence syntheses, from developing a protocol to reporting the review and planning an update. This follow-up paper considers reasons and practical implications for integrating a complex systems perspective in such reviews. This commentary describes how a complex systems perspective can benefit research, including evidence synthesis related to the CDoH, by embracing the complexity of the real world, understanding interventions in context, rendering visible the corporate playbook, and exposing the power dynamics that drive inequity. We then reflect on the practical implications of adopting a complex systems approach in CDoH evidence synthesis, including drawing on a systems lens and/or incorporating specific systems methods in the review. This commentary complements existing CODES guidance in highlighting considerations for conducting CDoH relevant evidence synthesis from a complex systems perspective. It can help interpreting such reviews and raise awareness of how commercial actors can shape evidence ecosystems, particularly evidence synthesis. Having a robust evidence base that considers systems elements and dynamics will support effective action to address the CDoH, improve public health and reduce inequity at scale.

## Introduction

The Commercial Determinants of Health (CDoH) are *“the systems, practices, and pathways through which commercial actors drive health and equity”* [1]. This recent definition, based on expert consensus, foregrounds the role of systems, with the authors explaining, “*[it] goes beyond a simple focus on unhealthy commodities and profits as the sole driver, instead recognising that the links between the commercial sector and health are varied, involving complex political, economic, and social systems”.* Adopting a complex systems perspective has been advocated when seeking to understand and address the CDoH and their impact on inequities [2].

Complex systems approaches are increasingly recognised as important for studying public health issues [3, 4]. A complex system has been defined as *“one that is adaptive to changes in its local environment, is composed of other complex systems (for example, the human body), and behaves in a non-linear fashion”* [5]. Complex systems approaches look beyond individuals and their behaviours to consider the dynamic and reflexive webs of social, cultural, political and economic structures they are caught up in [2]. They shift our focus from conceptualising pathways between interventions and outcomes in linear terms, towards understanding effects (or their absence) in the wider context, and considering the feedback loops and often unpredictable consequences of interactions within a system. However, such approaches have been critiqued as sometimes being vague, abstract and confusing [3]. For example, what does adopting a complex systems perspective entail for those undertaking CDoH evidence synthesis?

In this commentary, we draw on our collective experience of evidence synthesis (including systematic reviews), complex systems and/or CDoH research to set out why and how to integrate a complex systems perspective into CDoH-related evidence synthesis. After providing a brief introduction to the CDoH and complex systems approaches, we describe how a complex systems perspective can support the conduct and interpretation of research into the CDoH. We then reflect on the practical implications of adopting such an approach in evidence syntheses. This commentary builds on the CODES (Commercial Determinants and Evidence Synthesis) methodological guidance that collated, described and explained the specific considerations that researchers undertaking CDoH relevant evidence synthesis need to be alert to [6]. We considered the intersection of the CDoH and complex systems and report the additional implications to those already described by the CODES guidance [6] organised by stage in the systematic review process.

### The development of the field of the commercial determinants of health

Around a decade after the term was first used by West and Marteau [7], the CDoH were showcased in a Lancet series [8]. The field has diversified rapidly from an initial interest in the growing rates of chronic disease fuelled by the consumption of unhealthy products, such as tobacco and alcohol [9] to include industries such as pesticides, gambling, social media, fossil fuels and firearms, though research in all these areas has a long history beyond their inclusion in the CDoH [10–14]. These are termed unhealthy commodity industries as their primary product causes significant health damage [1]. The examination of the impacts of commercial activities has also extended to a wider range of health issues, including mental health and infectious disease [15–17]. The CDoH are also known to drive health inequities [18, 19], for example those experienced by Indigenous populations [12] or living in low- and middle-income countries [1, 20].

The commercial determinants operate at—and across—multiple levels that span from individual behaviour to structural factors [21], including meso-level business, market and political practices, and macro-level processes such as globalisation and climate change [22–25]. Building on the public health progress in countering the tobacco industry’s practices, advances have been made in identifying – if not yet fully evaluating—mechanisms through which CDoH might be addressed [22, 26]. Efforts have also been made to produce unifying definitions, frameworks and agendas for future CDoH research and practice [1, 27–29].

The opportunities – and necessity for – evidence synthesis in the field of CDoH is growing. This is partly due to a diversifying evidence base that is explicitly concerned with the commercial determinants. It is also driven by interest in systematically reviewing the wider body of evidence that relates to CDoH but is not explicitly named as such. It entails the drawing together of insights from different methodologies in order to better understand underlying commercial drivers of (ill) health—for example, factors influencing alcohol consumption and the prevention of alcohol-related harms [30]. Collating evidence from across industries can also be used to identify highly consistent practices- sometimes described as the “industry playbook” [31–34] – that directly and indirectly harm public health.

### Complex systems approaches

We now turn our attention to consider complex systems approaches in more detail. Table 1 – from McGill et al. [30]—defines the components and properties of a complex system. From a CDoH perspective, we suggest adding commercial practices to the list of system elements described in Table 1: these are at the core of Gilmore et al.’s definition, and relate to the political, scientific, marketing, supply chain and waste, labour and employment, financial and reputational management practices via which commercial actors exert their influence [1]. An example of scientific practices – and particularly relevant to this commentary on evidence synthesis—is how commercial actors seek to influence evidence ecosystems. These are networks of interactions – including the flow of information—among different organisations and people involved in the production, translation, and use of evidence such as scientific research [35, 36].

**Table 1:** Components of a complex system and related concepts, based on [30]

Concept	Definition
Elements	Components within a system (multiple ‘agents’, institutions, resources, commercial practices etc.)
Boundaries	The ‘limits’ or bounds of a given system; boundary judgements may be made by system actors (first order) or researchers (second order)
Levels	The structure of the system: levels may operate horizontally and/or vertically depending on boundary decisions
Relationship and interactions	Connections between different system elements, within and across system levels, and between elements and the broader context
Local rules	The norms and principles that guide interactions between system elements and drive system behaviour
Perspectives	The different ways actors within the system may view the system, their goals, their actions, and boundary decisions
Non-linearity	Inputs into a system may lead to a non-correspondingly sized impact
Feedback	Responses that either amplify (positive feedback) or dampen (negative feedback) the impacts of an intervention and may alter the intervention itself
Adaptation	The ways in which system elements and the system as a whole respond to internal and external outputs
Emergent properties	The emergent, collective behaviour of a system that cannot be reduced to its individual parts
Co-evolution	The changes to a system and the broader system in which it is located, over time
Unintended consequences	Processes and impacts that were unanticipated at the design stage of an intervention
System trajectories	The evolution of a system over time, which is path dependent or constrained in some ways due to its history

Complex systems approaches may be in tension with traditional research methods – specifically systematic reviews – that often seek to assess the effects of discrete interventions on one or more pre-identified outcomes through the synthesis of data, from a linear and somewhat decontextualised perspective while “controlling” for other variables and pathways. Instead, under a complex systems framing, public health interventions, including policies or services, can be thought of as system modifications [37] or “events within systems” [38].

Similarly, complex systems approaches differ from complex interventions as the latter often focus on a bundle of activities which are applied to a target population. Complex system thinking recognises that effects/outcomes are produced through interactions between a system’s agents and its context [39]. Outcomes are innate parts of the system, influencing its current form as well as being emergent properties of it*.* In this sense, “*Instead of asking whether an intervention works to fix a problem, researchers should aim to identify if and how it contributes to reshaping a system in favourable ways”* [4], p.2602).

As in primary research, a systems approach can be adopted in CDoH-related evidence synthesis. Using systems perspectives to synthesise disparate forms of evidence, which are commonly employed in the field of CDoH, are thought to be increasingly important by experts in the field as research and policy interest in the area grows [28]. Furthermore, ensuring that a CDoH lens is adopted when conducting evidence syntheses is critical to ensuring that the work does not reproduce industry-favourable framings and narratives, or generates biased findings by failing to consider the many ways in which industry influence can distort the evidence base/evidence ecosystem being reviewed [37]. Having robust evidence of the upstream commercial drivers of health and equity, and the direct and indirect pathways by which they act, will support effective action to catalyse positive change at scale.

## Why adopt a complex systems approach when reviewing evidence on the Commercial Determinants of Health?

The benefits of adopting a complex system approach when researching the CDoH and conducting evidence synthesis in this field include embracing the complexity of the real world, understanding interventions in context, rendering visible the corporate playbook, and exposing the power dynamics at play. These are described below. They can help to reveal and explain the resulting inequalities in health outcomes, to render visible factors that shape the system (e.g., the ways in which commercial actors follow the corporate playbook of practices to create and maintain systems favourable to their interests) and to inform potential ways to enact change.

### Embracing the complexity of the real world

Narrower, linear public health perspectives tend to frame commercial determinants as a subset of the social determinants of health, which may result in them being perceived as too diffuse and complex to understand, let alone tackle [21]. Commercial actors also echo such framings because they help to deflect attention from their activities onto wider aspects of ‘the system’. Conversely, systems approaches embrace this complexity and use it to understand a problem by identifying how, where and why it is caused within the system (e.g. by exploring how marketing drives harmful consumption). By making explicit the interconnections within and between such an understanding supports the identification of novel leverage points on which to intervene in order to improve public health [40]. Thus, while industry interests may prefer either to define problems as so simple that structural factors are irrelevant, or as so complex as to be insolvable, systems approaches point to the possibility of favourable change: they recognise that improvements in population health can be prompted by making small changes in multiple system elements [4]. The entire system does not need to change in order to improve public health, as illustrated by the impacts of minimum unit pricing in Scotland [41] and the sugar sweetened beverage tax in Mexico [25].

### Interventions in context

A core advantage of adopting a systems approach is producing a contextualised understanding of the effects of interventions, which can inform the framing of research questions in evidence syntheses. Interventions might be initiated by public or private actors. Here, as a case study, we draw on the alcohol industry’s deployment of the concept of “peer pressure” as a driver of underage drinking as a diversion tactic to avoid scrutiny of its own market-expanding activities. It is used to contrast a linear perspective with a complex systems perspective, in order to highlight the implications for evidence synthesis.

From a “traditional” systematic review perspective, if a researcher was reviewing the evidence on peer pressure as a determinant of underage drinking, they might include only studies of the effects of peer pressure on consumption [42]. Similarly, if concerned with evaluations of peer pressure interventions to reduce alcohol consumption, evidence from studies with the relevant outcomes would be included. However, this approach involves observing the intervention in isolation from its wider context, including other proximal and distal factors influencing underage drinking such as the diffuse, multiple and cumulative ways by which industry seeks to normalise alcohol consumption across society through fostering a more “alcogenic” environment [43]. A systems perspective takes a broader perspective which includes a wider range of processes, effects and changes in. Recognising these complex webs which shape behaviour – of both commercial actors and individuals—has important implications for undertaking CDoH evidence synthesis.

A tight focus on a small part of a system can benefit industry interests. For example, industry-funded research on peer pressure—including under-age drinking interventions which draw on the concept—are intended to distract from the role of alcohol advertising and marketing, and to shift responsibility from the industry to the individual (young people and their peers). Meanwhile, the promotion of these interventions by industry also assists in being able to present themselves as responsible public health or educational actors, thus helping to secure their place at the public health policymaking ‘table’.

An evidence synthesis which adopts a narrow perspective and considers a single type of intervention may even help to promote industry causes, particularly when industry actors have shaped the evidence base by commissioning primary research on their favoured interventions [44]. For example, unhealthy commodity industries often argue for, and fund, individual-level interventions (e.g. relatively ineffective interventions such as alcohol industry ‘responsible drinking’ campaigns [19, 45–47], and gambling industry ‘education’ campaigns [19, 48, 49]. These activities help to shape the evidence base, and policy discourse, in ways that “crowd out” or displace more effective and equitable population-level interventions which may affect profits (e.g., restrictions on marketing) [50, 51]. Therefore, there may be a misalignment between interventions with the largest evidence base – that appear to warrant being the subject of an evidence synthesis – and interventions which could have the greatest impact on public health.

Interventions can be promoted to divert attention onto areas which do not curtail harmful industry practices and thus may be intended to deflect scrutiny of industry commercial and marketing activities. For example, when Wood et al. [52] analysed email correspondence between a vice-president of The Coca-Cola Company and public health figures regarding the International Congresses of Physical Activity and Public Health in 2012 and 2014, they found that the former discussed with Coca-Cola sponsored researchers which specific topics to present at the conferences in an effort to shift blame for the rising incidence of obesity and diet-related diseases away from its products onto physical inactivity and a narrative of “individual choice” [52]. Similar diversionary tactics have been observed via the “safe storage” interventions promoted by the pesticides industry [12], and technological fixes such as carbon capture promoted by the coal and oil industries [14], which divert attention from pesticides and the need to reduce extraction and consumption of fossil fuels, respectively. Industry funding of researchers and academic institutions can itself be considered an intervention, one which “healthwashes” or “greenwashes” the industry whilst normalising academic-industry collaborations [40, 53]. In her book Unsavoury Truth, Marion Nestle describes how food science students at Cornell University take classes in the PEPSICO auditorium [54].

Industry actors may also misleadingly emphasise the apparent main outcome of their practices to divert attention away from other and/or wider effects on the system, which may in fact be the main desired outcome. For example, low-alcohol products may reduce consumption for individuals who previously did not drink at harmful levels, while simultaneously increasing population level exposure to alcohol brands by circumnavigating regulations, so-called “alibi marketing” [55–57]. Carlsberg’s marketing of low alcohol products during the UEFA EURO 2016 championship resulted in up to 358.6 million alcohol impressions which were delivered to children aged four to 17 years old during the final seven matches [58]. A review that focused narrowly on alcohol consumption outcomes in low-alcohol drinkers would miss this purpose, and wider, harmful, effects of such products and marketing strategies. A further feature of a complex systems approach is the ability to explore system adaptivity including unanticipated or unwanted outcomes following public health intervention. An example here is anticipating industry responses to the introduction of the soft drinks levy [59].

Whilst some industry-favoured interventions may be associated with statistically significant effect sizes, the magnitude of impact can be limited when considering clinical significance or when compared to more upstream or structural interventions [60]. Furthermore, any positive impacts of such interventions can be distributed unevenly between groups and are at risk of exacerbating health inequities. For example, school alcohol-awareness campaigns have demonstrated limited effectiveness in changing drinking behaviour [61] and may even cause harm through misinformation, for example through their opacity regarding the increased cancer risk associated with alcohol consumption [62]. The ethics – and opportunity costs—of deploying such interventions are highly questionable [63].

Interventions which can be effective at a consumer level may raise ethical issues or may be harmful when their balance of benefits and harms is considered at a population level. For example, when compared to traditional cigarettes, vaping may reduce – but not negate—individual level harm from carcinogens (whilst exposing the user to other less well evaluated toxins) [64, 65]. However, at a wider system level, these products are used strategically by the tobacco industry to target young people [66] and may institutionalise a different form of addiction [67].

In summary, a systems perspective is an analytical approach that moves progressively up from individual level impacts towards a consideration of structural factors such as influences on social norms. The broad perspective that this approach brings may add value when synthesising evidence regarding the wider systemic impacts of the CDoH.

### Rendering visible the corporate playbook

Assuming a complex systems vantage point can assist in identifying ‘corporate playbook’ strategies repeated across powerful industries to stifle public health controls and/or increase the sales of their health harming products [34]. These strategies act as brakes that help to maintain the status quo and ensure the resilience of systems which can promote poor health in the face of challenges from the public health community [37]

Working across industries to identify these strategies has been recognised as a productive approach: for example, in revealing competing interests in industry-linked education schemes [68], the complex and multiple interactions between private and public sector actors [34], commonalities in the use of evidence [32], and the ways in which they establish legitimacy and normalise their presence in decision-making fora [69].

### Evidence ecosystems and the commercial determinants of health

Efforts to manipulate the decision-making by using misinformation and disinformation are recognised as a growing threat to democracy and society [70, 71]. Industry also seeks to exert its influence through evidence ecosystems including shaping evidence production via funding research, research agenda setting [72], disseminating selective or (mis)information to members of the public via product labels and health education resources [73–75], and the lobbying of politicians and policy makers [76]. For example, the pharmaceutical industry has been active in shaping evidence ecosystems to focus on particular conditions and designing clinical trials to favour specific products, in part through comparator selection and outcome switching [77, 78]. Together, these activities help to shape prevailing discourses and framings of social norms surrounding their products, associated public health issues, and the possible policy responses in multiple and subtle ways [26]. As described above, commercial actors prioritise research that perpetuates narratives of putatively responsible consumption, skewing the evidence base—and policy attention—away from effective structural actions such as policies that limit marketing and availability of harmful products [79], a form of policy substitution [80]. Connecting the dots through a systems approach can allow for deeper analysis that reveals the implicit arguments and positions adopted by industry [59, 81].

### Power dynamics and inequalities

Complex systems thinking can help to extend public health’s frequent focus on individual level interventions addressing behaviours by rendering visible the influence of corporations that stretch through time and space, far beyond the moment of ‘unhealthy behaviour’ [2, 82]. Systems approaches can be applied to address contexts of marginalisation, conflicts, ethical issues and power relations [83] (see the section below on critical systems approach for further details). These challenges often characterise CDoH research which engages with the conflict between commercial and public health goals; the ethical issues of (for example) working with unhealthy commodity industries, and delegating responsibility to them to deliver, or partnering with them in the delivery of, health interventions [84, 85].

Explicit incorporation of corporate power in analyses—for example by considering the origins, nature and manifestations of connections between actors—has been advocated to better understand the CDoH, and improving public health [86], for example by considering corporate engagement with—and influence over—political institutions. [87]. A systems approach examining the commercial determinants can help to redress our collective blindness to the role of corporate power in creating and perpetuating poor health and health inequalities [24, 82]. The COVID-19 pandemic, economic downturns and racial inequity have highlighted the urgency for doing this [88, 89]. Analysis has revealed that the racialised marketing of unhealthy foods in the USA arises from an intersection of business models, marketing strategies of segmenting and targeting of specific population groups against a prevailing backdrop of structural racism [90]. Further upstream, the governance of key ultra-processed food corporations has been found to be dominated by large hedge fund managers who prioritise short-term financial returns and stifle public health-related shareholder proposals [91, 92].

With its concern regarding interactions, unintended consequences and feedback loops, complex systems approaches are well suited to thinking through the inadvertent – and crucially advertent—impacts on equity caused by the CDoH. The reach of multinational corporations spans the globe and is largely built on historical legacies including those of colonialism, discrimination, and marginalisation [27, 93]. Shareholders may be far removed—both socially and geographically – from the health harming effects of production, transportation and consumption, driving health inequity within and between populations [94]. For example, a systems-type approach has been used to synthesise the evidence on the mechanisms that enable the tobacco industry to externalise risks and costs away from high income countries to low- and middle-income countries, thus producing systematic tobacco-attributable inequalities [95]. Such a perspective can help to avoid reductionist and fragmented policy responses when seeking to address the supply of unhealthy commodities that fuel the incidence of non-communicable diseases around the world [96].

Systems thinking is helping to render visible the many ways corporate actors exert their power through their influence on clinical services and treatments. Even in countries such as the UK, with a dominant public healthcare sector, systems thinking is helping to elucidate the pathways by which pharmaceutical and diagnostic companies shape the framing of the causes of—and responses to—complex health problems such as antimicrobial resistance [16, 97]. Systems thinking has been applied to better understand the interactions between interventions seeking to alter antibiotic prescribing (a driver of antimicrobial resistance) and the wider health system thus revealing a complex and interacting set of proximal and distal factors which can have unpredictable effects [98, 99]. Furthermore, a better understanding of the social, economic, historical and political context can help to challenge the prevailing idea that antibiotic ‘misuse’ is predominately a problem of patients and doctor behaving inappropriately [98, 100]. In doing so, the portfolio of possible policy responses is broadened beyond educational and/or behavioural interventions.

## Implications for evidence synthesis

Below, we describe some implications of a systems approach when synthesising the evidence relevant to the CDoH. This builds on the CODES guidance and together are mindful of the range of methodological approaches implied in taking a complex systems perspective to knowledge.

In terms of operationalising systems perspectives in evidence synthesis, Hong et al. [83] identified two categories of reviews, both relevant to this article. The first group used established systematic review methods, including meta-analysis, but drew on a ‘systems lens’ to frame and/or organise their work. The second category used specific systems methods to investigate the connections between system components and develop ‘systems models’. In practice, the two categories may not always be completely separate; an evidence synthesis taking a holistic approach might develop a systems model in order to provide the ‘lens’ through which other types of research are examined [101].

### Using a ‘systems lens’

When using a systems lens, reviewers are able to employ many of the approaches used in systematic reviews following relatively conventional methods, such as those described in the Cochrane Handbook for Intervention Reviews [102]. The systems lens is variously used to identify variables, formulate hypotheses, develop a search strategy, inform study inclusion, guide the analysis, and interpretation of results. Reviews with a systems focus often require the incorporation of heterogenous study types [83]. This has important implications for how the review itself is conducted.

### Review questions and scope

Industry actors influence systems of evidence production and dissemination, both in the scientific literature and in the general media in order to promote their interests. Throughout the review process, therefore, the research team including Patient and Public representatives should be encouraged to critically reflect on how their own understanding/framing of the topic may have been shaped by what they have read, experienced and by circulating narratives [79]. Is there a possibility that the review may inadvertently reproduce industry-friendly framings and/or foreground industry-favoured interventions? Are there other ways to conceptualise the ‘problem’ and potential policy responses? How have health inequities been engaged with?

Petticrew et al. [39] provide examples of research questions to ask when seeking to understand interconnections within a system. Not all the aspects of the system, as described in Table 1, may be relevant to the research question. Table 2 illustrates how aspects of complex systems map onto review questions and eligibility criteria when applied to the CDoH.

**Table 2:** Operationalising systems approaches in Commercial Determinants of Health (CDoH) evidence syntheses. (Adapted from Petticrew et al. [39])

Aspect/component of complex system	Why is this of interest	CDoH example research question	Implications for evidence synthesis design
What ‘is’ the system, and how can it be described?	This can inform the scope of the review	What are the main influences on underage drinking? How are they created and maintained? How do they interconnect?	Scope of the inclusion and exclusion criteria – e.g. defining bounds of system Drafting a systems map that can be refined during the project may be an important part of developing the review protocol
Interactions between components of complex interventions	To gain a better understanding of which components are effective (some components may dampen effects)	What are the independent and combined effects of components of a complex intervention seeking to reduce underage drinking?	Quantitative and qualitative insights from studies with multiple arms are needed. Qualitative Comparative Analysis might be undertaken
Interactions of interventions with context and adaptations	Interventions may have context specific effects	How and why does the implementation of an intervention to reduce underage drinking vary by context? Does the effectiveness of the intervention differ by context?	Evidence from process mapping, case studies and modelling studies may be needed
How does the system change (systems adaptivity)?	Systems may accommodate or assimilate interventions	How does the alcohol industry respond to public health interventions?	Longitudinal data/Analysis of industry documents
Emergent properties	What properties emerge from synergistic qualities in the system	How does underage access to alcohol change following enhanced retailer inspections?	Qualitative studies may reveal unanticipated changes
Non-linearity and phase changes	Where the effect (or magnitude of the effect) does not seem directly related to the cause	How are changing gender norms altering alcohol consumption patterns?	Longitudinal data – need to ensure adequacy of follow up duration
Positive and negative feedback loops (reinforcing and balancing respectively)	This can potentiate or reduce the effects of interventions	What explains the change in effectiveness of the intervention over time. Are the effects dampened/increased by contextual factors (other system components)?	Qualitative and modelling studies may reveal these kinds of dynamic changes within systems
Multiple health (and non-health) outcomes	System changes can result in a range of intended and unintended consequences with no single ‘primary outcome’	What changes in the processes and outcomes happen following the system change? At what level of the system do they occur?	Tightly defined outcomes may miss unintended consequences

Defining the bounds of the system and the review scope impacts the search strategy**.** The use of ‘purposive’ searching, sometimes involving iteration may be recommended, rather than running a single, all-encompassing, search [103]. It should be borne in mind that comprehensive searching is only necessary when the review question is focussed on assessing effects of a specific program or policy. When working to develop systems models, finding *every* study that essentially contributes the same conceptual relations may be less important than ensuring that sufficient breadth in perspective is achieved. It may take several iterative cycles of developing the research question, running preliminary searches, refining the research question, running updated preliminary searches to find a happy medium between a search with appropriate breadth, and one that does not result in unmanageable numbers of records to examine.

### Using logic models

It can be helpful to produce a graphical representation of the concept of interest considering, in particular, how sectors and factors might interact to produce or prevent an outcome. Logic models are often split into two categories: “process-based” and “systems-based” [104]. While ‘process-based’ logic models can be useful when the review is focused on the interaction of a specific single intervention and its context, a ‘systems-based’ logic model will be more useful when using a systems lens as the analytic framework, since it emphasises understanding the dynamics of the wider system [105]. This helps to shift the review’s perspective from impacts on the individual to incorporate consideration of upstream factors and is often of great value when synthesising the evidence regarding the CDoH.

Developing a logic model in the earliest stages of a review, including in the protocol, can be a helpful tool to clarify the starting point – and assumptions – of the research team. The model can then be revised as the project progresses, with a finalised iteration reflecting the ultimate body of evidence synthesised by the review [6].

The value of cross disciplinary thinking is increasingly recognised when studying the CDoH [106]and drawing from fields beyond public health—including law, management and the political economy—can bring a richer body of evidence and expertise when developing the initial logic model. Preliminary literature searches in both academic and grey literature databases and consulting experts can provide additional cross disciplinary insights. Furthermore, involving people with lay experience of the product or topic of interest, including those who experienced harm, can help to challenge circulating narratives and the review team’s views, as well as identify aspects of the phenomenon that are under-evaluated. For example, group model building exercises with young people to explore the drivers of obesity identified causal pathways not previously considered by the research team [107].

It may also be useful to draw upon existing conceptual frameworks to situate the review question in its wider commercial, social and political context [6]. A number of CDoH frameworks have been developed including that of the Lancet Series [1]. Care is needed in the use of socio-ecological models, not only because they tend not to incorporate a systems perspective, but also because they may overlook or not explicitly articulate the role of commercial drivers/actors within their frameworks [108].

### Setting the eligibility criteria for including studies in the review

Hong et al. [83] highlight how reviews with a systems focus require the incorporation of a wide range of study types to inform the development of conceptual models. Given their interest in the strategies of commercial actors which create health harming systems and environments, CDoH reviews that adopt a systems perspective are similarly likely to require input from a wide range of types of evidence. Evidence syntheses provide the opportunity to bring together insights from a wide range of topics and/or using different types of data that might be difficult to do in primary research. For example, a systematic review of public–private partnerships aiming to improve food environments included studies involving humans, studies of products and places characteristics, and studies of documents about the partnerships themselves [109].

Furthermore, the evidence needed to document a ‘system’ may go beyond ‘traditional’ academic research. 

Examples of ‘non-traditional’ types of CDoH research include analyses of internal industry documents obtained through Freedom of Information requests [110]. These are used to reveal the typically unseen ‘backroom’ activities of health harming industries which may be in tension with their carefully curated public facing image. ‘Non-traditional’ data sources used to analyse ‘frontroom’ activities include analyses of purportedly health promoting apps [111] and the content of TikTok [13, 112].

Preliminary investigation into the practices and strategies of commercial actors within the (sub) system of interest will inform the types of evidence and study design relevant to the research question (Table 2), where the evidence is drawn from (e.g., which databases are searched) and inform the inclusion and exclusion criteria of the review.

### Selecting outcomes

Reviewers may adopt a systems perspective because they are interested in understanding at what level(s) a phenomenon/intervention operates and how their effects interact [39]. Therefore, relevant outcomes may be measured at different levels including at the individual level (e.g., product consumption), the family/household level (e.g., household spending), the community level (e.g., violence), and at the societal level (e.g., mortality, job market). In addition to outcome measures, reviews may also be interested in the processes (the mechanisms within the system) by which the changes observed occur [113].

The interest in multiple levels can be in contrast to industry’s focus on individual level outcomes which may not have the greatest health or policy importance [29], such as knowledge levels following information provision. Working with patient and public representatives and policy makers to identify relevant outcomes that are important to them can complement those identified from the literature, particularly if industry has successfully influenced research agendas and priorities in that field.

Any review of interventions should extract data on unintended outcomes, including adverse effects or harms [6]. Unhealthy commodity industries highlight potential unintended consequences of public health interventions as part of lobbying activities seeking to prevent or delay their implementation. These are repeated across industries: Arguments include that the proposed intervention will push consumers into riskier behaviour in unregulated or unlicensed spaces, have an inequitable impact/burden on certain population groups, and cause economic harm such as job losses [114]. Pre-specifying these within the review protocol, where relevant, will help to assess the veracity of these claims. Even when no relevant data are located, it enables push back against the certainty with which these claims are made [115].

### Risk of bias in the studies being synthesized

Whilst current risk of bias and quality appraisal tools may require the reporting of the presence of declared competing interests in primary studies or reviews, the tools do not necessarily require downgrading studies based on these risks. For example, in an umbrella review on vaping among young people, the only systematic review included that received a ‘high’ confidence rating using the tool AMSTAR 2 had clear industry links with a tobacco multinational [116]. This illustrates the need to treat conflicts of interests like the other risks of bias, and that industries can ‘tick the boxes’ in a risk of bias or quality appraisal tool in order to present their research as high quality.

Identifying potential conflict of interests beyond the information reported in studies in the authors’ affiliations, funding and declarations of interest statements is also challenging, such as those in research and professional networks, quid pro quo arrangements and conflicted affiliations. More detailed methods for assessing corporate involvement are in development [117, 118]. They include appraising Conflicts of Interest and Funding Statements within manuscripts and examining the authors’ employment and funding histories [119]. A limitation of these techniques is their dependence on collating insights from publicly available sources and time-consuming nature. It is important to bear in mind that whichever one is used, these tools appraise studies on an individual level or case-by-case basis, and do not identify more structural commercial influence, for example through the shaping of circulating narratives or steering the evidence base in a particular direction.

Reporting standards for patient and public representatives on declaring their engagement with industry is particularly underdeveloped. Contacting CDoH researchers who are experts on – but crucially independent from—specific industries may be able to alert you to problematic but outwardly appearing legitimate front groups, charities and research organisations, or undeclared interests of researchers and organisations.

There is also a need to develop and validate tools for study designs that are not traditionally included in evidence syntheses, such as studies of products, places, advertising and other environmental features, as well as studies of documents.

### Synthesis

While it is possible to use a systems lens as a way of, for example, undertaking meta-analysis or meta-regression, Hong et al. found only one example of a meta-analysis among reviews using this perspective [83]. More commonly used methods included narrative, realist, thematic, meta-synthesis, and content analysis. Thus, it may be that the use of a systems lens is associated with more interpretative methods of synthesis. Such as those described in the Cochrane-Campbell Handbook for Qualitative Evidence Synthesis [120].

Integrating findings from different types of study (and research paradigms) within a single review can be challenging, requires epistemological diversity and grappling with causal complexity, as summarised in Table 3.

**Table 3:** Key tenets of causal complexity [105, 121]

Causal complexity: Defined as comprising the states of:
• Conjunctural causation: Multiple factors or conditions come together to bring about an effect; they are seen as interdependent and complementary • Equifinal causation: There is not necessarily a single set of conditions that obtain the outcome. The same outcome may result from several different configurations of conditions • Asymmetric causation: Causal conditions that obtain an outcome do not mirror those conditions not obtaining the outcome. ‘That specific factors explain success does not imply that their absence leads to failure.’ [121]

Identifying the evidence that can shed light on a causally complex system involves careful consideration of how different types of evidence can contribute, and recognition that the methods usually held to provide causal security in systematic reviews are unlikely to be available. For example, the causal model underpinning a meta-analysis is well-established and accepted, with randomized trials generally recognised as providing the strongest grounds for causal claims. There are good reasons for this, but as it is improbable that any trials investigating CDoH have been conducted, other sources of evidence will be required. Moreover, the implications of causal complexity summarised in Box 1 require alternative ways of identifying causes, as many statistical methods of analysis cannot model conjunctural, equifinal or asymmetric causation adequately (especially given the relatively limited numbers of studies available for analysis).

Thus, as well as other types of quantitative evidence, it is likely that reviews of CDoH will also need to draw on qualitative research within their causal models. While qualitative research is often held to be incapable of demonstrating causal relationships and considered suitable only for ‘hypothesis generation’, there are many situations where causal claims rooted in qualitative (or ‘mechanistic’ or ‘theoretical’) evidence are widely accepted. Methods, such as qualitative comparative analysis, are designed to bridge the qualitative-quantitative divide and address the analytical complexities outlined in Box 1 [121, 122]. However, this is an active area of methodological exploration and development, so adaptation and innovation in methods regularly occurs in order to solve analytical challenges in the course of conducting reviews [123].

### Developing a ‘systems model’

A more radical departure from conventional systematic reviews are those approaches that seek to develop systems models. There is of course, some overlap with the approaches discussed above, where the dynamics of complex systems need to be accounted for in the synthesis. The main difference may be understood as a shift in perspective, between a situation where the impacts of CDoH are seen with respect to a specific set of outcomes (and interventions), to one which seeks to understand how the outcomes themselves are one aspect of a wider system, with potentially numerous implications for understanding how interventions might operate. Based on the ideas of Reynolds [124], Hong et al. organised reviews which developed systems models into three main approaches—hard, soft and critical (Table 4).

**Table 4:** Main types of systems approaches used in evidence synthesis (from [83, 89])

Approach	Ontology	Epistemology	Purpose	Examples of Methods
Hard	Realism	Postpositivism	Control Enables making sense of relationships between elements	Agent-based modelling Network analysis System dynamics
Soft	Relativism	Constructivism, interpretivism	Appreciation Enables communication and engaging contrasting perspectives	Inquiring system design Strategic assumption surface testing Soft systems methodology
Critical	Relativism	Constructivism, critical idealism	Emancipation Enables reconciliation of ethical issues and power relations	Community operational research System of systems methodologies Systemic intervention

Modelling systems dynamics was the most frequent of the ‘hard’ approaches identified by Hong et al [83]. This can involve bringing together insights from the evidence to initially formulate the problem and define concepts. Subsequently, the literature can be reviewed to define system boundaries, to identify system components and the links between them. Together, these support the development of causal loop diagrams, initial system models that map variables and their connections, and help refine initial hypotheses. For example, Sawyer et al. [125] created system maps to visualise the dynamics of the complex food environment underlying dietary intake in low-income groups based on their literature review findings. Similarly, Waterlander et al. [126] mapped the reviewed literature as an initial step in an integrated programme to address obesity-related behaviours in youth to help identify where actions could be developed. In their work on taking a systems approach across a research project, Knai et al. [127] refer to the importance of conducting foundational literature reviews to understand the context and history of a system of interest, and how and why implementation of a research activity might vary across contexts.

The literature can also be used to determine parameter values in simulation models as well as sourcing data to test, validate and calibrate models. Sensitivity-type analyses can be used to model the effects of different policy options [83].

Social network analysis can be used to map the actors involved and the connections between them, as has been done for stakeholders involved in multi-organisational initiatives seeking to transform the global food system [128] and alcohol industry authors [118]. Causal loop diagrams can also be generated to assist in visualising how different variables in a system are linked [129–131].

The next approaches identified by Hong et al. were ‘soft’ approaches, which can, for example, help identify responses to public health problems through exploring how meaning is constructed and the implications of different perspectives within a system. These approaches draw on more constructivist schools of thought and can use, for example, soft systems methodology, where accounts of different actors in the system are understood in their historical context, and where the analysis can result in a ‘meta narrative’ where historical ‘stories’ are described in relation to the phenomena of interest [132, 133].

The final group of reviews identified by Hong et al. adopted a critical approach and shared three underpinning concerns. These were: being critical about the chosen methods/theories (critique); freeing individuals and systems from oppression and coercion (emancipation); and using a combination of methodologies (pluralism). Hong et al. found that critical systems approaches were used to explore marginalization, ethical issues, and power relationships. As described above, these concerns run through much CDoH research [82, 113].

Blanchard et al. [134] created an evidence map to document the policy and evaluation characteristics of nearly 500 publications evaluating the effectiveness, cost-effectiveness, development and implementation of policies aiming to improve the food environment worldwide. This process allowed a critical visualisation and analysis of inequities in the origin, design, and scope of the evidence, and highlighted the lack of evaluations of health equity across the reviewed literature. This also illustrates the importance of thinking through whose perspectives/experiences are included in the literature and whose are excluded. Certain population groups may be systematically excluded, and this may not reflect the burden of harm experienced.

It can be difficult to describe complex systems in a linear narrative and figures will play a crucial part in communicating an overview of the findings. Furthermore, online, interactive systems maps – such as that describing the links between housing and health produced by the SIPHER project [135] can include summaries of the supporting evidence and reach a wider audience than academic articles in print.

### Planning the update of the review

In most research paradigms, best practice is not to update a review unless the evidence base has changed, partly to avoid research waste. However, CDOH researchers may need to operate more intentionally and in contravention to other research practices in this area. This is because the corporate playbook includes (a) dismissing older evidence (b) saying the evidence may have changed, or (c) saying ‘more research is needed’. Therefore, it may be warranted to repeat CDoH relevant reviews in a more rigorous manner even without key changes to the evidence base [39]. This might entail answering the same research questions but from a complex systems vantage point in order to more thoroughly investigate and examine industry claims.

## Conclusions

This commentary builds on the CODES guidance and considers the value and implications of undertaking evidence synthesis at the intersection of the CDoH and complex systems approaches [6]. This is important because public health is increasing its consideration of the importance of complex systems,recognising their value for both describing, understanding and analysing problems, and for identifying solutions (e.g. how might we intervene in systems to prevent harm [4]. Meanwhile, the most recent conceptualisations of the CDoH (i.e. the models produced by the Lancet Series [1] clearly characterise them as systems problems.

To address public health problems—as with other issues—we require rigorous evidence syntheses, but these need to evolve and be flexible to cope with emerging conceptual and methodological challenges, such as those outlined by Hong et al. [83]. In doing so, the payoff will be that syntheses of evidence on actions to address CDoH are better able to identify effective structural actions to modify systems in sustained, equitable and beneficial ways. This article is a step along this path.

The article is not without its limitations, in particular its methodological approach. There is an urgent need for guidance for the conduct of evidence syntheses within the field of the CDoH developed from a more systematic examination of the existing evidence and also based on consensus from a wider range of actors working in this field, including journal editors.

This article also contributes to thinking through how industry actors influence ecosystems of evidence. Whilst it is acknowledged that a cast of actors are involved in the production and dissemination of public health information, industry players have not been explicitly named within this [35]. This is despite the identification of the “Science for Profit model” within the field of the CDoH which describes the strategies used to maximise the amount, apparent trustworthiness, dissemination and uptake of industry-favourable science, while minimising these same aspects of industry-unfavourable science [72]. Recognising that unhealthy commodity industries influence ecosystems of evidence is a first step towards gaining broader acknowledgement of this and building momentum to address this outside of the CDoH community.

Consolidating systems approaches for evidence synthesis for the CDoH will help address imbalances in evidence driven by the actions of unhealthy commodity industries, with their well-documented preference for individual level responses that do little to threaten their business models.

 There will always be a need for both systems and non-systems approaches to reviews of the evidence prompted by a requirement for analyses and solutions at the system and process levels; however, one should not crowd out the other as doing so runs the risk of wasting resources and producing evidence syntheses favourable to industry but potentially harmful to public health.

## Data Availability

Data sharing is not applicable to this article as no datasets were generated or analysed during the current study.

## Abbreviations

CDoH: Commercial Determinants of Health
CODES: Commercial Determinants of Health and Evidence Synthesis
